# Isolation of *Haematococcus lacustris* as source of novel anti-multi-antibiotic resistant microbes agents; fractionation and identification of bioactive compounds

**DOI:** 10.1016/j.btre.2022.e00753

**Published:** 2022-07-08

**Authors:** Osama M. Darwesh, Rehab H. Mahmoud, Sayeda M. Abdo, Diaa A. Marrez

**Affiliations:** a Agricultural Microbiology Department, National Research Centre, Cairo 12622, Egypt; b Water Pollution Research department, National Research Centre, Cairo 12622, Egypt; c Food Toxicology and Contaminants Department, National Research Centre, Cairo 12622, Egypt

**Keywords:** *Haematococcus lacustris*, N-Hexane extract, GC–MS identification, Multi-antibiotic resistant microbes, Antibacterial activity

## Abstract

In this work, freshwater microalga, *Haematococcus lacustris* was isolated from the River Nile, identified and deposited in genebank under name of *H. lacustris* isolate REH10 with accession number OK336515. N-hexane extract was produced high inhibition effects against multi-antibiotic resistant pathogens. The n-Hexane extract was fractionated and 2 fractions (F3 & F4) exhibited high antibacterial activity (15 – 20 mm) compared with other fractions. Thus, they sub-fractionated and 2 sub-fractions produced from the F3 had high inhibition activity against all tested pathogens (18–20 mm). To identify the main compounds responsible for inhibition growth of multi-drug resistance bacteria, GC–MS chromatogram analyses was applied on the F3 and its sub-fractions 2 and 3. Five compounds detected in the 2 sub-fractions. Palmitic acid was identified as the first report antibacterial agent. The antioxidant activity of SF3–3 was reached to 86 and 80.5% for DPPH and ABTS.^+^ tests, respectively.

## Highlights

• Isolation and identification of *Haematococcus lacustris*.
• Production of novel Anti-Multi-antibiotic resistant microbes agents.
• Fractionation and sub-fractionation of n-Hexane microalgal extract.
• Identification of most active compounds using GC-MS.
• Evaluation of antioxidant activity of the produced compounds.

## 1 Introduction

Since antibiotics are losing their effectiveness at alarming rates due to the emergence of antibiotic-resistant microorganisms, bacteria have become more resistant to antibiotics, which have become a significant concern for public health [1], [2]. One of the biggest issues in healthcare today is treating infectious diseases, particularly those brought on by resistant bacteria. Major bacterial species have developed their resistance system to antibiotics; some of these species are now anticipated to be resistant to most or all antimicrobials, leading to "antibiotic resistance emergency" [3], [4]. Because of the widespread bacterial resistance to current antibiotics, it is crucial to discover novel bioactive chemicals. Microalgae are one potential source of novel antibacterial substances [5], [6]. Microalgae are common photosynthetic organisms found in a wide range of harsh habitats. They are extremely significant because they contain a variety of biologically active substances, including complex organic compounds and both primary and secondary metabolites, including vitamins, tannins, carbohydrates, terpenoids, phenolic compounds, phytopigments (xanthophylls and carotenoids), polyunsaturated fatty acids (PUFAs), peptides and docosahexaenoic acid (DHA), among others [7], [8], [9], [10]. In the search for novel antibiotic compounds needed to combat antibiotic resistant organisms that affects the efficacy of traditional medicines against bacterial infections in humans and animals, microalgae have been targeted species [11], [12], [13], [14]. These microorganisms have acquired defense mechanisms to tolerate exposure to fungi, bacteria and viruses [13], [15].  Microalgae extracts have been tested on cultures of pathogenic microbes. Fatty acids, tannins, phenolic substances, indole, terpenoids, polysaccharides, halogenated hydrocarbons, acetogenin, alkaloids, amides and inhibitory enzymes have been linked to the antibacterial action of these extracts [16], [17], [18].

*H. lacustris* is a single-celled freshwater biflagellate green alga of the class Chlorophyceae, order Volvocales, and family of Haematococcaseae [19], [20]. *Haematococcus lacustris* (previously named *Haematococcus pluvialis*) is regarded as the most hopeful microalgae for production of natural astaxanthin [21]. It is well acknowledged for its outstanding capability in synthesizing bioactive molecules with a wide range of biological activities, including like carotenoids, proteins, and fatty acids (FAs), in particular astaxanthin, a powerful antioxidant [22]. Hence, the purpose of our study was to explore novel antibacterial capacity of the *H. lacustris* extracts to fight some of dangerous pathogenic microbes.

## 2 Material and method

### 2.1 Microalga species isolation, purification and identification

Two locations on the River Nile bank provided the water samples that were used to isolate the microalgae. The first site was Ismailia Canal in the front of Port Said water plant Intake. The second site from the main river stream at Rosetta branch at the El-Rahawy drain discharged. Water samples (10 mL) were inoculated in 50 mL sterilized BG11 (Table 1) in a 100 mL conical flask under constant shaking (100 rpm) in a light incubator (Temp. 25 ± 2 °C, Light intensity ≈2500 lux).

**Table 1:** BG11 nutrient medium composition.

Macronutrient	mg/L	Micronutrient	mg/L
NaNO_3_	1500.00	H_3_BO_3_	2.86
K_2_HPO_4_	40.00	MnCl_2_·4H_2_O	1.81
MgSO_4_·7H_2_O	75.00	ZnSO_4_·7H_2_O	0.222
CaCl_2_·7H_2_O	36.00	Na_2_MoO_4_·2H_2_O	0.39
Citric Acid	6.00	CuSO_4_·5H_2_O	0.079
Na_2_CO_2_	20.00	Co(NO_3_)_2_·6H_2_O	0.0494
Na_2_EDTA	1.00		
Ferric Ammonium Citrate	6.00		

After visible mixed algal growth on liquid BG11 media, 100 µL of the mixtures were spread onto a BG11 agar plate under the same growth conditions. Repeated streaking onto fresh agar supplemented with BG11 and microscopic observations were applied until monospecific microalgae cultures were obtained individually and separately. Once, pure microalgae isolates were obtained singly, they were morphologically characterized. Genera of the isolates were identified by microscopic observation (Olympus CX41, equipped with an Olympus digital camera (Olympus SC30, Japan)) and the morphological characteristics noted [23]. The classical identification of the isolated microalgae was confirmed using molecular biology technique as previous described with slightly modifications [24]. The fresh microalga biomass was dried using liquid nitrogen for cell wall broken and then the buffer of sucrose-lysozyme (20 mg/ml) and proteinase K (1 mg/ml) was added on dried samples. The purification procedure on the total genomic DNA was done using isopropanol buffer [25]. Amplification reaction of the 16S rRNA gene was carried out by thermal cycler (Biorad, USA) using the extracted DNA with two primers F: (5′d AGAGTTTGATCCTGGCTCAG 3′) and R: (5′d TACGGTTACCTTGTTACGACTT 3′). The PCR conditions were adjusted according to Darwesh et al., [26]. For sequencing, the PCR product was purified using a QIAquick Gel Extraction Kit produced by QIAGEN, USA. The BLAST program was used to identify the received sequences (National centre for Biotechnology Information). Jukes Cantor Model was used to align the sequences and the neighbor-joining (NJ) approach was used for the phylogenetic reconstruction, along with bootstrap values, and the results were submitted to GeneBank.

### 2.2 Production of H. lacustris biomass

At optimal growth, the algae biomass was harvested by centrifugation at 5000 rpm for 15 min. Then, after removal of supernatant, the pellet of algae was rinsed several times by distilled water till the effluents became almost transparent. The washed biomass was freeze-dried at −50 °C and pressure <0.06 mbar for 6 h using a freeze-dryer (Christ, Germany) under vacuum conditions. The green dried biomass was stored at 4 °C until required for extraction.

### 2.3 Algal extract protocol

The crude methanol (70%) or n-hexane extraction from *H. lacustris* was performed using ultrasonic-assisted protocol. The crude extraction was prepared at biomass concentration of 10 mg/mL. The mixtures after incubation for overnight were sonicated by ultrasonic water bath (Elmasonic S 60H, Germany) performed at power and frequency of 150/550 W and 37 kHz, respectively for several times till exhaustion. The extracts were collected, filtered and the filtrate was evaporated till dryness, using rotatory evaporator, under reduced pressure, for distillation of the solvent.

### 2.4 Fractionation and isolation of bioactive compounds using column chromatography

By using column chromatography, the chloroform extraction of n-hexane extract was fractionated. Briefly, 30 g of silica gel (0.06–0.2 mm, 70–230 mesh ASTM) was added to a glass column (30 × 500 mm) together with 5 g of anhydrous sodium sulfate to generate a slurry. Chloroform served as the carrier solvent. In order to let the silica gel packing settle while the extra chloroform was exhausted, the stopcock was opened. To stop the silica gel column from drying up during draining, an additional 5 g of anhydrous sodium sulfate was added to the top of the gel.

The column was loaded with 100 mg of chloroform extract in 10 ml, and the flow was set to one drop per second. The order of solvent of elution in column chromatography was fixed as chloroform: methanol (98:2), (95:5), (90:10), (80:20), (50:50), (25:75) and finally methanol 100% to give 7 fractions. The column fractions (50 mL each) were evaporated under vacuum and analyzed for antimicrobial activity. The active fractions in antimicrobial assay were divided to sub-fractions by submitted to column chromatography to develop by its elution and divided to 5 sub-fractions (10 mL each).

### 2.5 GC–MS identification of bioactive compounds

Thermo Scientific's Trace GC Ultra / ISQ Single Quadrupole MS, TG-5MS fused silica capillary column was utilized for the GC–MS analysis (30 m, 0.251 mm, 0.1 mm film thickness). Helium gas was employed as the carrier gas at a constant flow rate of 1 ml min^−1^, and an electron ionization apparatus with ionization energy of 70 eV was used for GC–MS detection. The temperature of the MS transfers line and injector was fixed at 280 °C. The oven's temperature was set to begin at 50 °C and hold it for 2 min before rising to 150 °C at a rate of 7 °C per min, 270 °C at a rate of 5 °C per min (hold it for two min), and then 310 °C at a rate of 3.5 °C per min as the final temperature (hold 10 min). Using a percent relative peak area, the quantification of all the discovered components was examined. Based on a comparison of the compounds' relative retention times and mass spectra with those of the NIST, WILLY library data from the GC–MS instrument, a preliminary identification of the compounds was carried out.

### 2.6 Antibacterial activity evaluation of H. lacustris hexanolic extract and its fractions

**Fig. 1:**
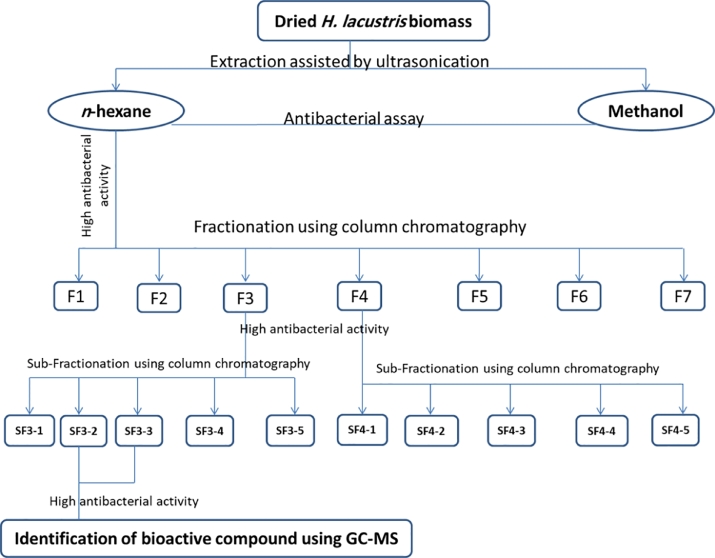
Flowchart of extraction, fractionation, sub-fractionation and identification of bioactive compounds from *H. lacustris*.

Crude n-hexane and methanol extract of *H. lacustris* as well as fractions and sub-fractions as appeared in Fig. 1 were used as antibacterial agents. The antibacterial activity was assessed using well diffusion technique according to the previous extensive procedures [27], [28]. It used against multidrug-resistant bacteria, Gram-positive like *Staphylococcus aureus* ATCC-47,077 (St.) and *Listeria monocytogenes* ATCC- 35,152 (List.) and Gram-negative bacteria like *E. Coli* ATCC-25,922 (EC.) and *Salmonella typhi* ATCC-15,566 (Salm.) in addition to *Candida albicans* ATCC-10,231 (C Alb.) as yeast model. Overnight culture of microbial suspension was adjusted to 0.5 McFarland equivalents (1.5 × 10^8^ CFU/mL). The dried surface of nutrient agar was inoculated with the examined pathogens by the swab and then 6 mm well was made and impregnated with 70 μL of subjected solution. The wells contained DMSO has been regarded as negative control. All plates had been incubated at 37 °C for 24 h. Erythromycin and Vancomycin have been performed as standard antibacterial agents against all pathogens. The zone of inhibition was measured (mm). Experiments have been performed in triplicate and numbers reported on average.

### 2.7 Antioxidant activity evaluation for fractions and sub-fractions of H. lacustris hexanolic extract

Antioxidant activity of fraction # F3 and its sub-fractions were determined using DPPH˙ and ABTS˙^+^ free radical scavenging assay described by Mohdaly et al., [29] with some modifications. For DPPH˙ test, 0.1 ml of the sample (0.1 mg lyophilized /ml DMSO) was added to 3.9 ml of freshly prepared DPPH solution (22 mg of DPPH in 50 ml methanol) after diluting to reach the absorbance value of 0.8 ± 0.02 at 515 nm. After vortexing for 30 s and incubation in the dark place for 30 min, the reaction mixture was measured at 515 nm using UV–Vis spectrophotometer (JASCO, A114761798, JAPAN). The DPPH free radical scavenging activity was calculated according to the following equation (Eq. (1)).

(1) $$\text{DPPH}\;\text{radical}-\text{scavenging}\;\text{activity}\;\left(\%\right)=\left[A_{b}-A_{s}/A_{b}\right]\times 100$$

Where (A_b_), the absorbance of the control reaction (without the tested sample) and (A_s_), the absorbance of the tested sample.

In case of ABTS˙+ assay, the ABTS˙+ solution was diluted with distilled water to obtain an absorbance of 0.70 ± 0.02 at 734 nm. A 0.1 mL of sample (0.1 mg lyophilized/ mL DMSO) was added to 0.9 ml of distilled water and 1 mL of ABTS˙+ solution and then incubated at room temperature for 7 min. The absorbance was recorded at 734 nm and ABTS˙+ scavenging effect (%) was calculated as follows (Eq. (2)).

(2) $$\text{ABTS}\;\text{radical}-\text{scavenging}\;\text{activity}\;\left(\%\right)=\left[A_{b}-A_{s}/A_{b}\right]\times 100$$

Where (A_b_), the absorbance of the control reaction (without the tested sample) and (A_s_), the absorbance of the tested sample.

### 2.8 Statistical analysis

The statistical significance of the tested treatments was analyzed using Statistica Version 9 (StateSoft, Tulsa, Okla., USA). The analysis of variance was mentioned as means (ANOVA, one way analysis) (*p* < 0.05). Followed by Least significant differences (LSD) method for comparing significant alterations between treatments.

## 3 Results and discussions

### 3.1 Isolation, identification and production of novel H. lacustris biomass

**Fig. 2:**
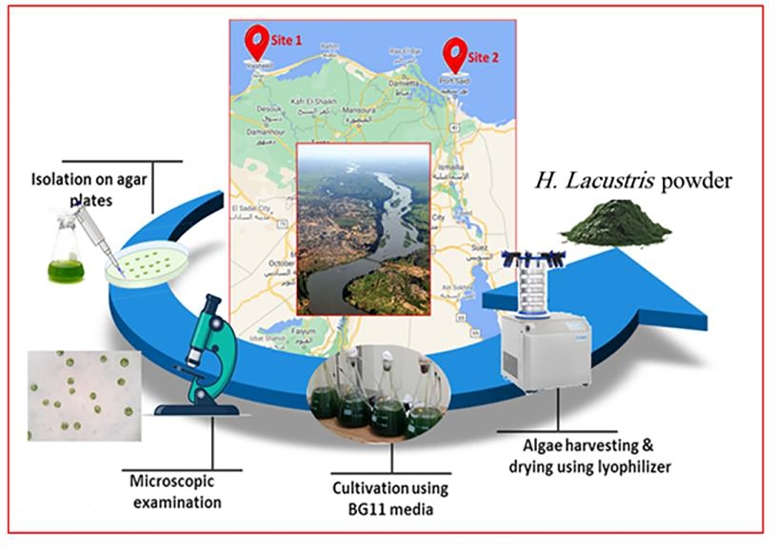
Flowchart for isolation and production of *H. lacustris* biomass.

**Fig. 3:**
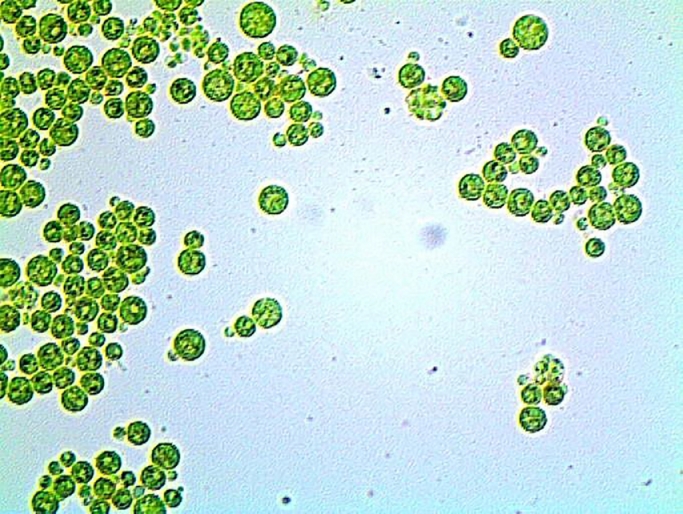
Light microscopy image of *Haematococcus lacustris* isolate REH10 (40X).

**Fig. 4:**
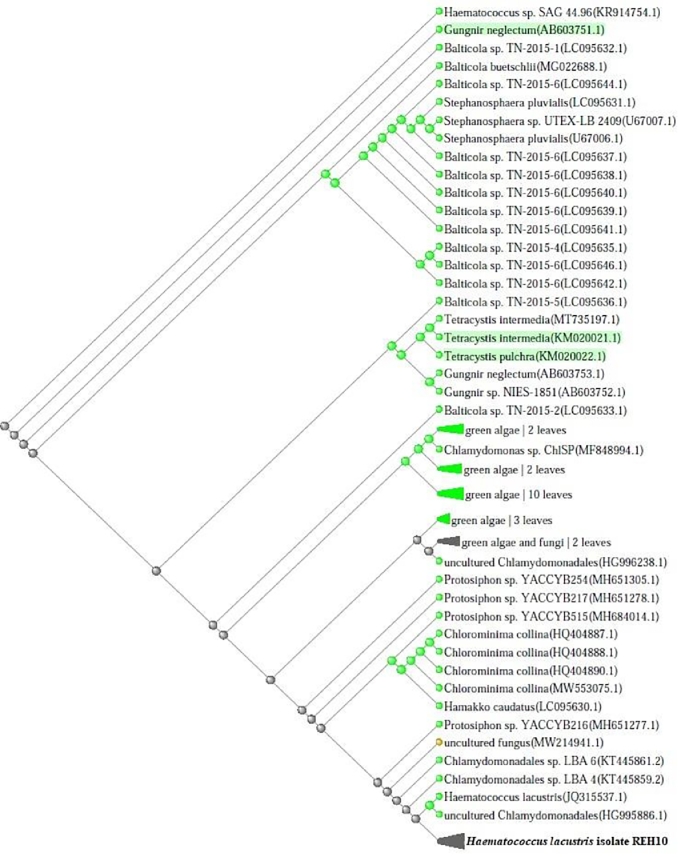
Neighbor joining phylogenetic tree constructed for the strain *Haematococcus lacustris* isolate REH10 with other NCBI strains.

Ten water samples (5 from each site) were collected and transferred to the Lab for isolation their content of microalgae. After purification and microscopic examination, one microalga species was obtained (Fig. 2). The purified microalga was cultivated using BG11 medium and subjected to identify using molecular biology procedures. Morphological characteristics of the isolates *H. lacustris* was presented in Fig. 3 as light microscopic observation. The microscopic examination showed that the microalga was unicellular green with thick-walled palmelloid. The results of sequencing showed that the obtained isolate was closely related to *H. lacustris* species and recorded in gene bank under accession number OK336515 with the name of *Haematococcus lacustris* isolate REH10 (Fig. 4).

The data in Fig. 4 represented the phylogenetic tree of the novel isolate with other related strains in gene bank. The genus *Haematococcus*, an unicellular freshwater bi-flagellates microalgae, was described over 150 years ago [30], but is still unknown to greatest biologists. Because it contains the keto carotenoid astaxanthin, which can build up in huge amounts inside lipid vesicles in the cytoplasm and is responsible for the red blood color [31], [32]. The *Haematococcus* genus is now understood with several species, but for the long time, only one species was formally documented. Given that it is frequently referred to by two different names, *H. lacustris* and *H. pluvialis*, the nomenclature of this species is unclear [30]. Currently, it appears that these two names are interchangeable, and according to Nakada and Ota [33], the right nomenclature for this strain is *H. lacustris.*

### 3.2 Antibacterial activity of H. lacustris hexanolic and methanolic extracts

**Fig. 5:**
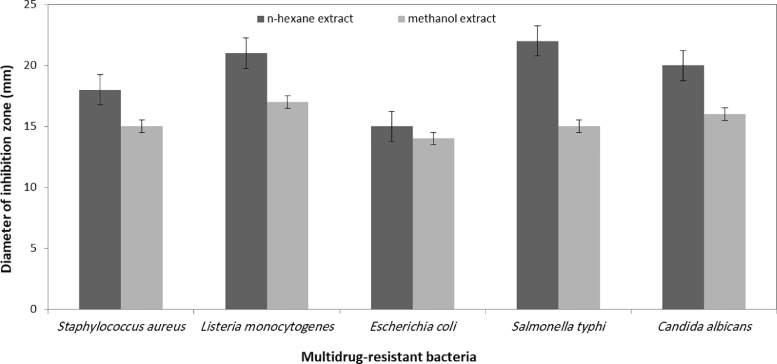
Anti-multidrug resistant bacteria *H. lacustris* n-hexane and methanol extracts.

Crude n-hexane or methanol extracts of *H. lacustris* were applied as anti-bacterial against multidrug-resistant bacteria. Due to resistance of such microbes to broad spectrum of antibacterial drugs, it is necessary to provide new agents with inhibitory activity against these pathogens [34], [35]. Microalgae as photosynthetic microorganisms have acquired the attention of scientists due to its vast potential applications such production of antimicrobial agents, anticancer materials, antioxidants and other bioactive compounds [36], [37]. Thus, the antibacterial activity of *H. lacustris* extracts was assessed using well diffusion technique according to the previous extensive procedures. Additionally, it has been noted that the growth inhibition of *B. subtilis, St. aureus*, and *E. coli* is caused by the short and long-chain fatty acids from *H. pluvialis* and *Scenedesmus obliquus* [27], [38]. In our study, n-hexane and methanolic extract of *H. lacustris* was evaluated its inhibition activity against different bacterial pathogens. The results stated that the antibacterial activity of the extracted fatty acids ranged between 18 and 22 mm of inhibition zone diameter and higher than methanolic extract (Fig. 5). For this reason, the crude hexane extract was fractionated.

### 3.3 Anti-multidrug resistant bacteria for fractions and sub-fractions of H. lacustris hexanolic extract

**Fig. 6:**
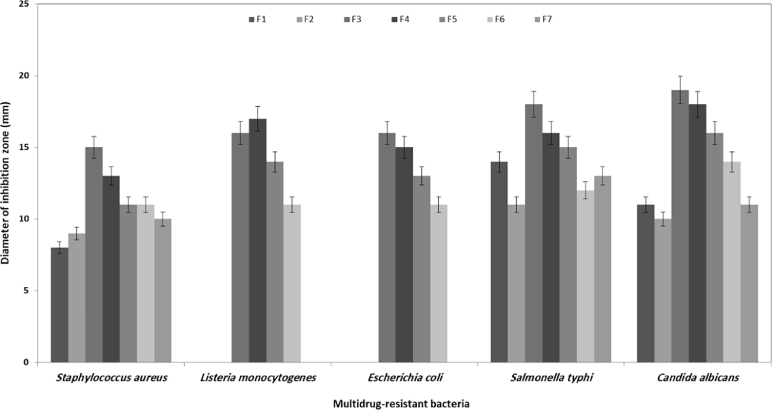
Anti-multidrug resistant bacteria for seven fractions of *H. lacustris* n-hexane extract.

Many bioactive substances were discovered from microalgae like carotenoids, fatty acids, and phycobiliproteins. Fatty acids or related lipid compounds are derivatives of hexanolic extracts [39]. Therefore, in the current study, seven fractions of *H. lacustris* hexanolic extract were produced and used as the source of antibacterial agents. The results illustrated in Fig. 6 showed that 2 fractions (3 and 4) had high antibacterial activity compared with other fractions. They had activity against the tested pathogens reached to 15 – 20 mm of inhibition diameter. In this study, we fractionated hexanolic extract to obtain the causer of antibacterial activity. In this regard, Gnanakani and his coauthor [40] stated that fatty acids produced by *Nannochloropsis oculata* exhibited inhibition activity against gram positive and gram-negative pathogenic bacteria. From the data obtained in this procedure, we can conclude the fractions number 3 and 4 contained high amounts and derivatives of bioactive fatty acids or lipids compounds. For that, these 2 fractions were sub-fractionated.

**Fig. 7:**
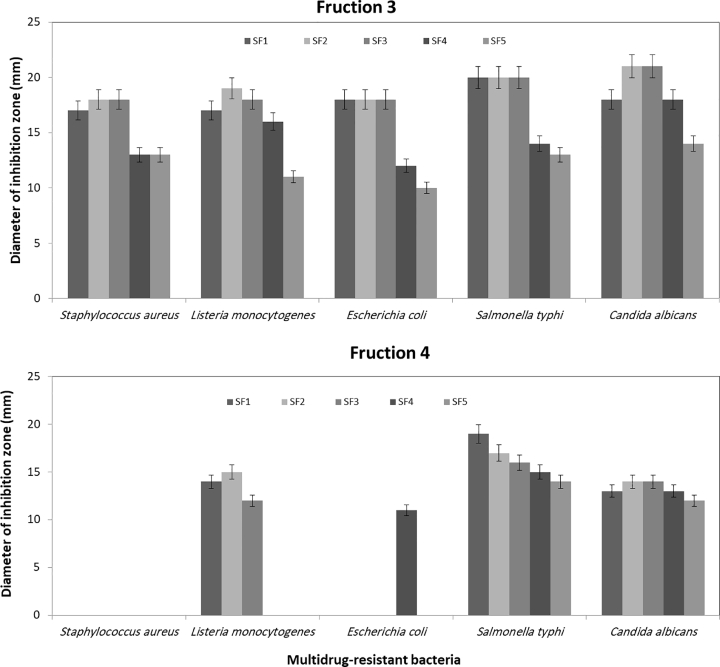
Anti-multidrug resistant bacteria for sub-fractions of *H. lacustris* n-hexane extract.

The fractions 3 and 4 produced by (90:10) and (80:20) chloroform: methanol mixture (v/v), respectively were sub-fractionated to provide the main compounds responsible for inhibition of microbial growth. The data illustrated in Fig. 7 showed that the sub-fractions 2 and 3 have high inhibition activity against all tested pathogens, they have inhibition diameter ranged between 18 and 20 mm. This led to these sub-fractions contained antibacterial compounds by large amounts compared with other sub-fractions. The hexane fractions are recognized to contain lipids and fatty acids substances [41]. Not all these fatty compounds had antimicrobial activity, thus, we try to focus about the active compounds against multi-drug resistant bacteria by sub-fractionation of highly active fractions. The produced sub-fractions were appeared to have almost the same activity of fractions. This means that only some sub-fractions contain the same bioactive compounds. Many papers have reported the antibacterial activity of several microalgae extracts and returned this activity to the presence of fatty acids. These fatty acids inhibited the growth of Gram-positive (including multidrug-resistant *Staphylococcus aureus*) and Gram-negative bacteria [42], [43]. Antibacterial activity of hexanolic extract fatty acids has also been reported for *Haematococcus pluvialis* [44]. For that, it is important to characterize and identify the main compounds responsible on inhibition growth of multi-drug resistance bacteria. To do that, GC–MS chromatogram analyses was applied on the fraction 3 and its sub-fractions 2 and 3.

### 3.4 Identification of bioactive compounds using GC–MS

**Fig. 8:**
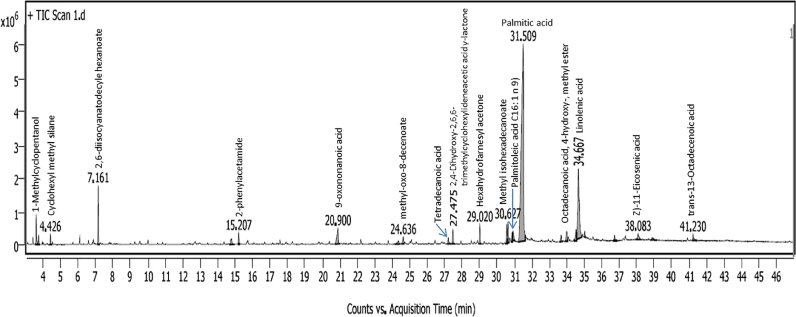
GC–MS chromatogram of fraction F3 from *H. lacustris* hexane extract.

Gas chromatography with mass spectrum was applied to identified the main components related to the most active fractions and sub-fractions of *H. lacustris* n-hexane extract and the resulted charts were illustrated in Fig. 8 and Table 2. Sixteen compounds were detected in fraction # 3 as the most active one against the multi-antibiotic resistant bacteria. After sub-fractionation, two sub-fractions coded SF3–2 and SF3–3 were recorded the high antibacterial activity. The data obtained from GC–MS showed that the SF3–2 involved 6 compounds out of 16 compounds detected in the main fraction, while the second sub-fraction (SF3–3) involved 9 compounds. Five compounds, Methyl isohexadecanoate, Palmitoleic acid, Palmitic acid, Linolenic acid and trans-13-Octadecenoic acid were detected in the 2 sub-fractions (Table 2). Microalgae as microbial plant is produced many beneficial materials varied from fatt acids, amino acids, enzymes, phenolic and flavonoids, …..etc. Such these compounds were used as antimicrobials, anti-cancers, anti-flamatories, antioxidants agents [27], [45]. Mubarak and his coworkers [46] noted that the fatty acid, 9-octadecenoic acid produced by hexanolic extract of *Scenedesmus bijugatus* had inhibition activity against *E. coli, S. aureus* and *C. albicans*. Also, many compounds were extracted by Marrez et al., [47] with various percentages like 3-hexadecyloxycarbonyl-5-(2‑hydroxyl)−4-methylimidazolium, 3‑hydroxy, dodecanoic acid 9,12,15-octadecadienoic acid, 9-octadecenoic acid (Z), 2-hexadecenal, 40-trimethoxy, quercetin and octasiloxane.

**Table 2:** Components detected in fraction 3, sub-fraction SF3–2 and SF3–3 of *H. lacustris* hexane extract.

Peak	RT	Component name	Area Sum%		
			Fraction F3	Sub-fraction SF3–2	Sub-fraction SF3–3
**1**	3.61	1-Methylcyclopentanol	1.78	–	–
**2**	4.43	Cyclohexyl methyl silane	0.75	–	–
**3**	7.16	2,6-diisocyanatodecyle hexanoate	3.89	–	0.41
**4**	14.8	2-phenylacetamide	1.08	–	–
**5**	20.9	9-oxononanoic acid	2.88	2.22	–
**6**	24.64	Methyl-oxo-8-decenoate	0.88	–	0.7
**7**	27.22	Tetradecanoic acid	0.69	–	–
**8**	27.47	2,4-Dihydroxy-2,6,6-trimethylcyclohexylideneacetic acid γ-lactone	1.32	–	–
**9**	29.02	Hexahydrofarnesyl acetone	1.51	–	0.43
**10**	30.63	Methyl isohexadecanoate	1.93	4.39	12.13
**11**	30.91	Palmitoleic acid C16: 1 n 9)	1.3	9.04	8.32
**12**	31.51	Palmitic acid	55.3	44.4	30.69
**13**	33.99	Octadecanoic acid, 4‑hydroxy-, methyl ester	1.8	–	0.41
**14**	34.67	Linolenic acid	14.02	38.33	40.73
**15**	38.083	Z)−11-Eicosenic acid	1.17	–	–
**16**	41.24	trans-13-Octadecenoic acid	0.85	0.35	0.73

The biological activity of fractions obtained from *H. lacustris* hexane extract was represented in Table 3. From the data noted in this Table showed many biological activity of detected compounds like antioxidant, antibacterial, anti-nociceptive, anti-inflammation, anti-thrombotic and biosurfactant activities. Also, we found that two compounds, Palmitic acid and Linolenic acid were detected as over 50% and had surfactant and antibacterial activity, respectively [48], [49].

**Table 3:** The identified common-components in the fractionation of hexanol extract and their biological activity.

Component name	Biological activity	References
9-oxononanoic acid	stimulate the activity of phospholipase A_2_	[53]
Methyl-oxo-8-decenoate	Antioxidant activity	[50]
2,6-diisocyanatodecyle hexanoate	Not studied	
Hexahydrofarnesyl acetone	antibacterial, anti-nociceptive and anti-inflammation activities	[54]
Methyl isohexadecanoate	Antibacterial	[55]
Palmitoleic acid C16: 1 n 9)	anti-thrombotic	[56]
Palmitic acid	Surfactant	[49]
Octadecanoic acid 4‑hydroxy- methyl ester	Antioxidant and antimicrobial activities	[50]
Linolenic acid	Antibacterial	[48]
trans-13-Octadecenoic acid	Antioxidant activity	[50]

In our study, Palmitic acid were detected as the highest bioactive compound in the fraction 3 and sub-fractions (2 and 3) also. This compound was recorded as the first report of antibacterial agent in the current study, this may be due to its biosurfactant activity. Because, the biosurfactant properties provide that this compound attached the surface of bacteria and blocked it from moving followed by lyses. Also, four compounds were noted as antibacterial agents (Table 3), however 3 compounds were reported as antioxidant substances as mentioned by Rahman et al., [50].

### 3.5 Antioxidant activity for fractions and sub-fractions of H. lacustris hexanolic extract

**Fig. 9:**
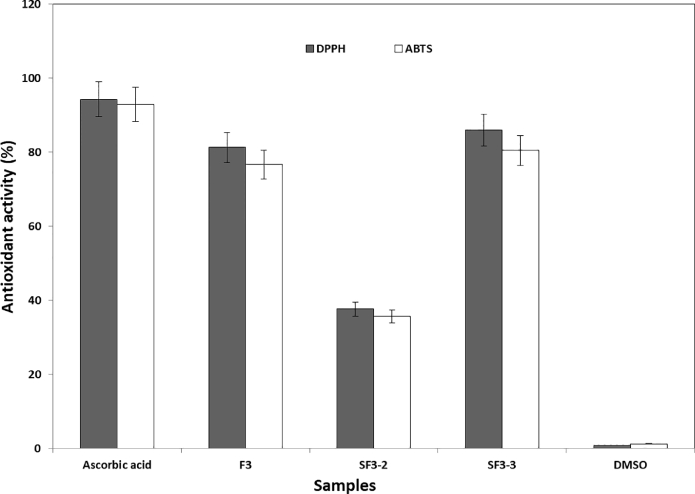
Antioxidant activity for fraction (F3) and sub-fractions (SF3–2 and SF3–3) of *H. lacustris* hexanolic extract compared with ascorbic acid (+ control) and DMSO (- control).

Depend on the data illustrated in Table 3, some detected compounds in fraction and sub-fractions of *H. lacustris* hexanolic extract had antioxidant activity as historical studies [50]. Therefore, we evaluated the antioxidant activity of the *H. lacustris* hexanolic extract either fraction or sub-fraction using DPPH and ABTS.^+^ radical cation assays and the results represented in Fig. 9. The results of antioxidant activity stated that fraction 3 of *H. lacustris* n-hexane extract had antioxidant activity about 81 and 76% for DPPH and ABTS.^+^ tests, respectively. This may be due to found some antioxidant compounds like trans-13-Octadecenoic acid, Octadecanoic acid 4‑hydroxy-methyl ester and Methyl-oxo-8-decenoate. These compounds were detected in the sub-fraction 3, this clarify the increase activity of this fraction compared by the second one (SF3–2), because these compounds were absent in the sub-fraction 2. The antioxidant activity of SF3–3 was reached to 86 and 80.5% for DPPH and ABTS.^+^ tests, respectively. The search to replace synthetic antioxidants by natural substitutes is developed daily depend environmental-safe properties of natural sources (Saranya et al., 2014). For that, we tried to provide new antioxidant and antimicrobial agents. Microalgae are noted as rich source of novel biological active metabolites either primary or secondary which have drawn attention of the pharmaceutical industries. At the scale of human cells, the reactive oxygen species (ROS) are designed by endogenous influences resulting in a wide oxidative damage and age related deteriorating conditions. In this regard, natural antioxidants can rapidly react with these ROS and delay the extent of oxidative decline [44], [51], [52]. Also, Abdel-Karim et al. [52] stated that different extracts of *C. vulgaris* showed a great variety of secondary bioactive compounds particularly flavonoids, alkaloids and phenols with antioxidant capacity.

## 4 Conclusion

*Haematococcus lacustris* as freshwater microalgae was isolated from the River Nile for production of bioactive compounds against some multi-antibiotic resistant pathogens. This isolate was identified and deposited in genebank under name of *Haematococcus lacustris* isolate REH10 with accession number OK336515. N-hexane extract of *H. lacustris* and its fractions and sub-fractions was produced inhibition effects against the tested pathogenic microbes. Also, Palmitic acid as a surfactant was identified in this study as the first report antibacterial agent.

## Funding

This research did not receive any specific grant from funding agencies in the public, commercial, or not-for-profit sectors.

## Declaration of Competing Interest

Authors declare that there are no conflicts of interest.
